# Contribution of the clinical information to the accuracy of the minimally invasive and the complete diagnostic autopsy^☆^^☆☆^

**DOI:** 10.1016/j.humpath.2018.10.037

**Published:** 2019-03

**Authors:** Fabiola Fernandes, Paola Castillo, Quique Bassat, Llorenç Quintó, Juan Carlos Hurtado, Miguel J. Martínez, Lucilia Lovane, Dercio Jordao, Rosa Bene, Tacilta Nhampossa, Paula Santos Ritchie, Sónia Bandeira, Calvino Sambo, Valeria Chicamba, Sibone Mocumbi, Zara Jaze, Flora Mabota, Mamudo R. Ismail, Cesaltina Lorenzoni, Ariadna Sanz, Natalia Rakislova, Lorena Marimon, Anelsio Cossa, Inacio Mandomando, Jordi Vila, Maria Maixenchs, Khátia Munguambe, Eusebio Macete, Pedro Alonso, Clara Menéndez, Jaume Ordi, Carla Carrilho

**Affiliations:** a Department of Pathology, Maputo Central Hospital, 1100 Maputo, Mozambique; b Faculty of Medicine, Eduardo Mondlane University, 1100 Maputo, Mozambique; c ISGlobal, Hospital Clinic, Universitat de Barcelona, 08036 Barcelona, Spain; d Department of Pathology, Hospital Clinic, Universitat de Barcelona, 08036 Barcelona, Spain; e Centro de Investigação em Saúde de Manhiça, 1100 Maputo, Mozambique; f ICREA, Catalan Institution for Research and Advanced Studies, Pg. Lluís Companys 23, 08010 Barcelona, Spain; g Pediatric Infectious Diseases Unit, Pediatrics Department, Hospital Sant Joan de Déu, Universitat de Barcelona, 8950 Barcelona, Spain; h Universidad Europea de Madrid, 28670 Madrid, Spain; i Department of Microbiology, Hospital Clinic of Barcelona, Universitat de Barcelona, 08036 Barcelona, Spain; j Department of Medicine, Maputo Central Hospital, 1100 Maputo, Mozambique; k Department of Pediatrics, Maputo Central Hospital, 1100 Maputo, Mozambique; l Department of Gynecology and Obstetrics, Maputo Central Hospital, 1100 Maputo, Mozambique; m Consorcio de Investigación Biomédica en Red de Epidemiología y Salud Pública (CIBERESP), 28029 Madrid, Spain

**Keywords:** Minimally invasive autopsy, Complete diagnostic autopsy, Resource-limited settings, Cause of death determination, International Classification of Diseases, Tenth Revision

## Abstract

Although autopsy diagnosis includes routinely, a thorough evaluation of all available pathological results and also of any available clinical data, the contribution of this clinical information to the diagnostic yield of the autopsy has not been analyzed. We aimed to determine to which degree the use of clinical data improves the diagnostic accuracy of the complete diagnostic autopsy (CDA) and the minimally invasive autopsy (MIA), a simplified pathological postmortem procedure designed for low-income sites. A total of 264 coupled MIA and CDA procedures (112 adults, 57 maternal deaths, 54 children, and 41 neonates) were performed at the Maputo Hospital, Mozambique. We compared the diagnoses obtained by the MIA blind to clinical data (MIAb), the MIA adding the clinical information (MIAc), and the CDA blind to clinical information (CDAb), with the results of the gold standard, the CDA with clinical data, by comparing the *International Classification of Diseases, Tenth Revision* codes and the main diagnostic classes obtained with each evaluation strategy (MIAb, MIAc, CDAb, CDAc). The clinical data increased diagnostic coincidence to the MIAb with the gold standard in 30 (11%) of 264 cases and modified the CDAb diagnosis in 20 (8%) of 264 cases. The increase in concordance between MIAb and MIAc with the gold standard was significant in neonatal deaths (*κ* increasing from 0.404 to 0.618, *P* = .0271), adult deaths (*κ* increasing from 0.732 to 0.813, *P* = .0221), and maternal deaths (*κ* increasing from 0.485 to 0.836, 0.;*P* < .0001). In conclusion, the use of clinical information increases the precision of MIA and CDA and may strengthen the performance of the MIA in resource-limited settings.

## Highlights

• The addition of clinical data increases the diagnostic accuracy of the minimally invasive autopsy (MIA) and complete diagnostic autopsy in 12% and 8% of the cases, respectively.
• The increase in concordance from MIA blind to clinical data to MIA enhanced with clinical data was significant in neonatal, adult, and maternal deaths and was also evident in children, although it did not reach statistical significance.
• The use of clinical data may improve the diagnostic precision of the MIA in resource-limited settings.

## 1 Introduction

The autopsy is the gold standard methodology for cause of death investigation. It is also a valuable tool to maintain accurate mortality statistics, which remain essential for public health and health service planning. Unfortunately, autopsy rates, which have markedly declined in Western countries in the last decades, have always been very low in low-income countries. Indeed, the feasibility of conducting autopsies in these sites faces notable barriers including cultural and/or religious apprehension, which lead to a poor acceptability of the conventional autopsy procedure [1], [2]. The lack of infrastructures and the low number of trained pathologists are also a limitation for a time-consuming examination such as complete diagnostic autopsy (CDA). Finally, the fact that many deaths occur outside the health system [3] results in a serious constraint for autopsy practice in low-income countries.

In the last few years, our group has developed and refined a minimally invasive autopsy (MIA) method specifically designed for low-resource sites as a feasible alternative to the CDA. The procedure, which consists of sampling of fluids and key organs using biopsy needle followed by histopathologic and microbiological investigation of the obtained samples, can be rapidly performed by trained technicians close to the place where death occurs and is more acceptable than the CDA [4]. Thus, MIA can be relatively easily implemented as a surveillance method in settings where cause of death information is scarce and most needed [5].

This MIA procedure has recently been validated for neonates, children, maternal deaths, and other adults from Mozambique [6], [7], [8], [9]. In these validation studies, the concordance between the 2 procedures (MIA and CDA) has been moderate for neonatal and maternal deaths (*κ* = 0.404 and 0.485, respectively) and substantial for children and adults (*κ* = 0.704 and 0.732, respectively). In all these previous studies, the MIA results have been analyzed blindly to any clinical data in an attempt to determine the accuracy of the technique per se. In contrast, in all these studies, the CDA diagnosis included, as it is the rule in the routine activity of all departments of pathology, not only a thorough evaluation of all available macroscopic and microscopic and microbiological results, but also any available clinical data [10].

The contribution of the clinical data to the diagnostic yield of the MIA has not been analyzed. Interestingly, to our knowledge, the degree to which the addition of the clinical data results in an increase in the diagnostic yield of the CDA has not been evaluated either. Thus, the aim of this study is to determine the contribution of adding the clinical information to the microbiological and pathological results of the MIA in the final attribution of cause of death. We have also evaluated the contribution of the use of the clinical information to the accuracy of the CDA. For this purpose, we have compared the diagnoses obtained with the sole laboratory evaluation of the samples acquired through the MIA and the CDA with those diagnoses achieved after the addition of the clinical information to the laboratory results.

## 2 Materials and methods

### 2.1 Study setting and design

This observational study received the approval of the Clinical Research Ethics Committee of the Hospital Clinic of Barcelona (Spain; File 2013/8677) and the National Bioethics Committee of Mozambique (Mozambique; Ref. 342/CNBS/13). The study was conducted at the Department of Pathology of the Maputo Central Hospital, a 1500-bed government-funded quaternary health care center, in collaboration with the departments of obstetrics and gynecology, pediatrics, and internal medicine.

Deaths of all ages (with the exception of neonates) having occurred in the hospital premises from November 2013 to March 2015 were eligible for recruitment if they fulfilled the following criteria: (1) a CDA requested by the clinician as part of the medical evaluation of the patient;, (2) a verbal informed consent to perform the autopsy given by the relatives;, and (3) no traumatic origin.

Coupled MIA and CDA procedures were performed in 264 cases. The study included 41 neonates (median age, 3 days; range, 0-25 days), 54 children (median age, 4 years; range, 1 month–15 years), 57 maternal deaths (median age, 27 years; range, 15-39 years), and 112 other adults (median age, 36 years; range, 15-76 years).

### 2.2 Autopsy procedures and laboratory analyses

Detailed pathological and microbiological methods of the MIA have been described elsewhere [11], [12]. All MIA procedures aimed at obtaining samples of the blood, cerebrospinal fluid, liver, lungs, bone marrow, central nervous system, heart, kidney, and spleen (and also the uterus and placenta, if available, in maternal deaths). We used a portable ultrasound (US) scan device (Mindray Z6; Mindray Med Int Ltd, Shenzhen, China) to evaluate the liver, the spleen, the kidneys, and, in women of childbearing age, the pelvis. Any lesions or abnormal fluid (ascites, pleural effusions) identified, as well as the position of the spleen and the kidneys, were recorded. After the US examination, the gel was removed, the areas of the body to be punctured were cleaned and sterilized, and the samples were obtained without direct US guidance [11]. Samples were obtained for microbiological and histologic analyses. The body fluids were analyzed only with serologic and microbiological techniques. Within 30 minutes after completing the MIA, the CDA procedure was performed by another pathologist not involved with the MIA following a standardized protocol [10], [13]. Histologic and microbiological analyses were conducted from the same viscera collected in the MIA and also from any gross lesions, when identified. No samples of the body fluids (blood and cerebrospinal fluid) were obtained at the CDA.

The histologic samples from the MIA were analyzed blindly to any clinical information by a team of 2 pathologists not involved with the CDA. Two microbiologists evaluated the results of the microbiological analyses. Details of the analytical methodology have been described elsewhere [6], [7], [8], [9]. The microbiological analyses included both classic cultures and molecular techniques.

After a washout period of 3 to 6 months, the same team of experts analyzed the samples of the CDA following the same approach used for the analysis of the MIA samples, but tissues obtained during CDA were not cultured, and only molecular methods were used to investigate pathogens. In the pathological evaluation, the macroscopic data of the CDA were available to the investigators, but the investigators were blind to the clinical information.

### 2.3 Review of the clinical charts

Clinical information from all recruited patients was collected using a standardized questionnaire. The same investigator (Q. B.) was tasked with conducting the clinical data abstraction of all cases after a thorough revision of the entire medical record. The data collection included, among others, retrieving from the clinical process demographic data, medical history, and information about the inpatient admission process and the clinical information of the disease during hospitalization including signs and symptoms, physical examination, laboratory results, imaging results, and treatment received. In maternal and neonatal deaths, the obstetrical history was also included.

### 2.4 Determination of the cause of death blind to clinical data

Once the analyses of the MIA samples had been completed, a panel evaluated the pathological and microbiological reports data of the MIA and assigned the diagnosis. This panel was composed of a pathologist and a microbiologist, as well as a pediatrician, an obstetrician, or a clinician with expertise in epidemiology depending on the age group of the patients evaluated in a session. In the first cause of death attribution meeting, the MIA diagnosis was made in the absence of the clinical information (“MIA blind,” ie, MIAb diagnosis).

Once the analyses of the CDA samples had been completed, a different panel composed of a pathologist, a microbiologist, and a clinician evaluated the pathological and microbiological reports of the CDA and assigned the cause of death using the same methodology. Similarly, in this first cause of death attribution meeting, the CDA diagnosis was made in the absence of the clinical information (“CDA blind,” ie, CDAb diagnosis).

### 2.5 Determination of the cause of death enhanced with clinical data

Twelve to 18 months after the MIAb diagnosis was obtained, a different panel with identical composition (1 pathologist, 1 microbiologist, and 1 clinician) reviewed again the MIA microbiological and histologic reports, this time with all available clinical data. Thus, a new MIA diagnosis enhanced with clinical information (MIAc diagnosis) was assigned.

After a minimum washout period of 3 months (range, 3-6 months), the same panel involved in MIAb analysis also evaluated the data from the CDA and assigned the final CDA diagnosis of cause of death using the same methodology. This final CDA diagnosis integrated all the findings from the autopsy (macroscopic, histologic, and microbiological analyses) and the clinical information (CDAc diagnosis) and was considered the gold standard for cause of death attribution.

### 2.6 Cause of death coding and diagnostic grouping, definition of concordance and coincidence in diagnosis, and definition of change in diagnosis

All morbid conditions identified in the MIAb, the MIAc, the CDAb, or the CDAc, directly leading to death and any underlying conditions (if present), as well as any other significant conditions possibly contributing to death, were codified following the *International Classification of Diseases, Tenth Revision* (*ICD-10*). This codification process was conducted independently for the MIAb, MIAc, CDAb, and CDAc. Although all diseases included in the chain of events leading to death (underlying conditions) as well as any significant conditions possibly contributing to death were evaluated, only the final cause of death, that is, the disease directly leading to death, was analyzed to compare the methods.

Diagnoses were grouped into broad classes, specifically defined for each study group [6], [7], [8], [9]. Neonatal deaths were classified into 6 classes (infectious diseases, intrapartum complications, preterm complications, congenital malformations, other conditions, and nonconclusive). Pediatric deaths were classified into 5 classes (infectious diseases, malignant tumors, congenital malformations, other diseases, and nonconclusive); adult deaths, 4 classes (infectious diseases, malignant tumors, other diseases, and nonconclusive); and maternal deaths, 6 classes (pregnancies with abortive outcome, hypertensive disorders, obstetric hemorrhage, pregnancy-related infections, nonobstetric complications, and unexplained deaths). The diagnostic concordance between 2 methods was determined by comparing the diagnostic classes. The diagnoses were considered as concordant when they were in the same diagnostic class.

The diagnostic coincidence between 2 methods was determined by comparing the *ICD-10* codes. The *ICD-10* system classifies diagnoses into nested categories of different hierarchical levels, where diseases or conditions are organized in chapters, blocks, and 3 character categories [14]. The diagnoses obtained with 2 methods were considered as coincident when they were identical in chapter and block.

Any variation in terms of block between 2 methods was considered as a change in diagnosis. Any case in which the addition of clinical data caused a change in the *ICD-10* coding resulting in a coincidence in terms of the *ICD-10* block with the gold standard was considered an increase in diagnostic accuracy.

### 2.7 Statistical methods

The concordance between the diagnostic categories obtained in the MIAb, MIAc, and CDAb with the gold standard (CDAc) was evaluated by means of the *κ* coefficient, which was interpreted as suggested by Landis and Koch [15]. The differences between the 2 *κ* values (MIAb versus CDAc and MIAc versus CDAc) were assessed based on Student *t* distribution of 1000 bootstrap replications of paired differences for comparing correlated *κ* coefficients [16]. The change in the proportion of cases with an identifiable cause of death (both by MIA and CDA) and in the overall agreement of the MIA with the gold standard when clinical information was added was assessed using the McNemar exact test. The statistical analyses were performed using Stata version 14.1 (Stata, College Station, TX) [17].

## 3 Results

### 3.1 Cause of death determination using MIA and CDA methods

A cause of death was identified in the MIAb in 235 (89%) of 264 cases, whereas in 29 cases (11%), no conclusive diagnosis was reached. After adding the clinical data, a cause of death was defined in the MIAc in 246 (93%) of 264 cases.

A cause of death was identified in the CDAb in 257 (97%) of 264 cases, whereas in 7 cases (3%), no conclusive diagnosis was reached. After adding the clinical data, a cause of death was identified in the CDAc in 263 (99%) of 264, and only 1 case remained as nonconclusive.

### 3.2 Concordance in disease grouping with the gold standard

The overall concordance of the MIAc with the gold standard regarding identical diagnostic class increased from 80% (212/264) to 89% (235/264; *P* < .001). The overall concordance of the CDAb with the gold standard was 250 (95%) of 264.

In neonatal deaths, the agreement in disease grouping between the MIAb and the gold standard was 68% (28/41 cases; *κ* value = 0.404, moderate agreement) and increased to 78% (32/41 cases; *κ* value = 0.618, substantial agreement) when including the clinical data in the MIA (comparison MIAc versus gold standard). The increase of *κ* value was statistically significant in this age group (*P* = .027).

In children, the agreement in disease grouping between the MIAb and the gold standard was 89% (48/54 cases; *κ* value = 0.704, substantial agreement), which increased to 93% (50/54 cases; *κ* value = 0.802, almost perfect agreement) in the comparison between MIAc and the gold standard. This increase of *κ* value did not reach statistical significance (*P* = .160).

In maternal deaths, the agreement in disease grouping between the MIAb and the gold standard was 68% (39/57 cases; *κ* value = 0.485, moderate agreement) compared with the gold standard. This value increased to 89% (51/57 cases; *κ* value = 0.836, almost perfect agreement) in the comparison between MIAc and the gold standard. The increase of *κ* value was statistically significant in this group ( 0.;*P* < .001).

In the adult group, the agreement in disease grouping between MIAb and the gold standard was 87% (97/112 cases; *κ* value = 0.732, substantial agreement), which increased to 91% (102/112 cases; *κ* value = 0.813, almost perfect agreement) in the comparison between MIAc and the gold standard. The increase of *κ* value was again statistically significant in this group (*P* = .022).

### 3.3 Changes in diagnostic coincidence related to the addition of clinical data

Overall, the addition of clinical data resulted in a change in the MIAb diagnosis in 35 of 264 cases; in 30 of them, the change resulted in a better coincidence with the gold standard diagnosis (increase in the diagnostic accuracy). Thus, the overall coincidence of the MIAb with the gold standard regarding identical diagnostic block was 155 (59%) of 264 and increased to 70% (185/264) for the MIAc diagnosis (*P* = .008). The overall coincidence of the CDAb with the gold standard was 244 (92%) of 264. The addition of clinical data significantly modified the CDAb diagnosis in 20 (8%) of 264 cases. In 17 of them, the MIAb to MIAc diagnoses had also changed, whereas in 3 cases, the clinical information had not resulted in a change in the MIAb diagnosis. Table 1 shows detailed information of the cases in which the addition of clinical information resulted in a change in diagnosis (*ICD-10* block) between the MIAb and the MIAc and/or between the CDAb and the CDAc (the gold standard).

**Table 1:** Changes in *ICD-10* cause of death diagnosis between the MIAb and the MIAc

MIAb		MIAc		CDAb		CDAc	
Diagnosis	*ICD-10* code	Diagnosis	*ICD-10* code	Diagnosis	*ICD-10* code	Diagnosis	*ICD-10* code
Neonates							
Respiratory syncytial virus pneumonia^a^, ^b^	J12.1	Sepsis of newborn (no agent)	P36.9	Sepsis of newborn (streptococcus, group B)	P36.0	Sepsis of newborn (streptococcus, group B)	P36.0
Pneumonia no agent^a^, ^b^	P23.9	Intestinal occlusion	P76.9	Intestinal occlusion	P76.9	Intestinal occlusion	P76.9
Unknown^a^	R99	Kernicterus, unspecified	P57.9	Kernicterus, unspecified	P57.9	Kernicterus, unspecified	P57.9
Respiratory syncytial virus pneumonia^b^, ^c^	J12.1	Severe birth asphyxia	P21.0	Respiratory syncytial virus pneumonia^b^	J12.1	Severe birth asphyxia	P21.0
Neonatal sepsis (*Escherichia coli*)^c^, ^d^	P36.4	Severe birth asphyxia	P21.0	Unknown	R99	Severe birth asphyxia	P21.0
Neonatal sepsis (*Klebsiella pneumoniae*)^e^	P36.4	Neonatal sepsis (*K pneumoniae*)	P36.4	Neonatal sepsis (*K pneumoniae*)^d^	P36.4	Pulmonary hemorrhage	P26.9
Children							
Pneumonia (*K pneumoniae*)^b^, ^f^	J15.0	Sepsis (*K pneumoniae)*	A41.5	Peritonitis (*K pneumoniae)*	K65.0	Peritonitis (*K pneumoniae)*	K65.0
Sepsis (*Streptococcus pneumoniae*)^c^, ^d^	A40.3	Hereditary factor VIII deficiency	D66	Intracerebral hemorrhage^b^	I61	Hereditary factor VIII deficiency	D66
Pneumonia (*S pneumoniae*)^b^, ^c^	J13	Tetanus	A35	Pneumonia (*S pneumoniae*)^b^	J13	Tetanus	A35
Pneumonia (*Haemophilus influenza*)^b^, ^c^	J14	Rabies	A82	Pneumonia (*H influenza*)^b^	J14	Rabies	A82
Unknown^c^	R99	Rabies	A82	Tuberculosis^b^	A15	Rabies	A82
Pulmonary hemorrhage^e^	R04.8	Pulmonary hemorrhage	R04.8	Severe pulmonary congestion and hemorrhage^b^	R04.8	Malaria	B50.9
Maternal deaths							
Sepsis (*K pneumoniae*)^a^, ^d^	A41.5	Hemorrhagic shock secondary to retained placenta	O72.0	Hemorrhagic shock secondary to retained placenta	O72.0	Hemorrhagic shock secondary to retained placenta	O72.0
Hepatic failure, unspecified^a^, ^d^	K72.9	Hemorrhagic shock secondary to rupture of uterus during labor	O71.1	Hemorrhagic shock secondary to rupture of uterus during labor	O71.1	Hemorrhagic shock secondary to rupture of uterus during labor	O71.1
Sepsis (*Enterobacteriaceae*), HIV+^a^, ^b^	B20.1	Puerperal sepsis	O85	Puerperal sepsis	O85	Puerperal sepsis	O85
Cytomegalovirus disease, HIV+^a^, ^b^	B20.2	Hemorrhagic shock postpartum secondary to vaginal laceration	O71.4	Hemorrhagic shock postpartum secondary to vaginal laceration	O71.4	Hemorrhagic shock postpartum secondary to vaginal laceration	O71.4
Unknown^a^	R99	Hemorrhagic shock secondary to abdominal pregnancy	O00.0	Hemorrhagic shock secondary to abdominal pregnancy	O00.0	Hemorrhagic shock secondary to abdominal pregnancy	O00.0
Suggestive of cardiovascular disease^b^, ^c^	O99.4	Hemorrhage due to labor complication (fetal macrosomy)	O67.9	Cardiovascular disease (heart hypertrophy)^b^	O99.4	Hemorrhage due to labor complication (fetal macrosomy)	O67.9
Puerperal sepsis^c^, ^d^	O85	Hemorrhagic shock secondary to premature separation of placenta	O45.9	Puerperal sepsis^d^	O85	Hemorrhagic shock secondary to premature separation of placenta	O45.9
Sepsis (*Enterobacteriaceae*)^c^, ^d^	A41.5	Hemorrhage due to premature separation of placenta with coagulation defect	O45.0	Sepsis (*Enterobacteriaceae*)^d^	A41.5	Hemorrhage due to premature separation of placenta with coagulation defect	O45.0
Sepsis (*Enterobacteriaceae*), HIV+^c^, ^d^	B20.1	Hemorrhagic shock secondary to placenta accrete	O72.0	Unknown	R99	Hemorrhagic shock secondary to placenta accrete	O72.0
Unknown^c^	R99	Hemorrhagic shock secondary to premature separation of placenta	O45.9	Intra-abdominal hemorrhage (postpartum)^g^	O72	Hemorrhagic shock secondary to premature separation of placenta	O45.9
Unknown^c^	R99	Hemorrhagic shock secondary to premature separation of placenta	O45.9	Unknown	R99	Hemorrhagic shock secondary to premature separation of placenta	O45.9
Unknown^c^	R99	Hemorrhagic shock secondary to uterine atony	O45.9	Unknown	R99	Hemorrhagic shock secondary to uterine atony	O45.9
Adults							
Unknown^f^	R99	Hypertension	I10	Cerebral hemorrhage	I61	Cerebral hemorrhage	I61
Unknown^f^	R99	Hypertension	I10	Cerebral hemorrhage	I61	Cerebral hemorrhage	I61
Cardiovascular disease, unspecified^f^, ^g^	I51.6	Hypertension	I10	Dilated cardiomyopathy	I42.0	Dilated cardiomyopathy	I42.0
Cardiovascular disease, unspecified^f^, ^g^	I51.6	Hypertension	I10	Acute myocardial infarction	I21	Acute myocardial infarction	I21
Cardiovascular disease, unspecified^a^, ^g^	I51.6	Cerebral hemorrhage	I61	Cerebral hemorrhage	I61	Cerebral hemorrhage	I61
Cardiovascular disease, unspecified^a^, ^g^	I51.6	Cerebral infarction	I63	Cerebral infarction	I63	Cerebral infarction	I63
Cardiovascular disease, unspecified^f^, ^g^	I51.6	Type 2 diabetes mellitus with ketoacidosis	E11.1	Diabetic and hypertensive nephropathy	E11.2	Type 2 diabetes mellitus with ketoacidosis	E11.1
Cardiovascular disease, unspecified^a^, ^g^	I51.6	Gastric ulcer with hemorrhage	K25.0	Gastric ulcer with hemorrhage	K25.0	Gastric ulcer with hemorrhage	K25.0
Alcoholic cirrhosis^a^, ^b^	K70.2	Esophageal varices with bleeding	I85.0	Esophageal varices with bleeding	I85.0	Esophageal varices with bleeding	I85.0
Pneumonia (*S. dysgalactiae*)^c^, ^d^	J15.3	Type 2 diabetes mellitus with ketoacidosis	E11.1	Pulmonary edema^g^	J81	Type 2 diabetes mellitus with ketoacidosis	E11.1
Unknownc	R99	Gastroenteritis of unspecified origin	A09	Unknown	R99	Gastroenteritis of unspecified origin	A09
Unknown^c^	R99	Gastroenteritis of unspecified origin	A09	Unknown	R99	Gastroenteritis of unspecified origin	A09
Unknown^c^	R99	Rabies	A82	Unknown	R99	Rabies	A82
Cardiovascular disease, unspecified^e^	I51.6	Cardiovascular disease, unspecified	I51.6	Hypertensive heart disease with acute pulmonary hemorrhage^g^	I51.6	Cardiac arrest, sudden cardiac death	I46

NOTE. For each case, the diagnosis of the CDAb and the gold standard (CDAc) are also included.

a Cases in which the clinical information resulted in an increased diagnostic accuracy between MIAb and MIAc with no changes in the diagnostic accuracy of CDAb versus CDAc.

b Cases in which the diagnosis reached in the MIAb or CDAb was included as an associated condition in the final chain of events of the gold standard.

c Cases in which the addition of the clinical information resulted in an increased diagnostic accuracy between MIAb and MIAc and also in an increased diagnostic accuracy between CDAb and CDAc.

d Cases in which the MIAb or the CDAb cause of death was considered to be a misdiagnosis due to an overinterpretation of microbiological and/or histologic findings with a low level of evidence.

e Cases in which the addition of the clinical information resulted in no change between MIAb and MIAc diagnosis, but it resulted in a change between CDAb and CDAc.

f Cases in which the addition of clinical data to the MIAb (MIAb to MIAc) did not result in an increased diagnostic accuracy.

g Cases in which the gold standard diagnosis was a refinement of the MIAb or CDAb diagnosis.

In neonates, the addition of clinical information resulted in a change in diagnosis from the MIAb to the MIAc in 5 (12%) of 41 cases. All 5 changes resulted in an increased coincidence with the gold standard diagnosis. In 2 of these cases, the improved coincidence with the gold standard diagnosis was also observed after the addition of clinical data to the CDAb diagnosis.

In the pediatric group, a change in diagnosis from MIAb to MIAc occurred in 5 (9%) of 54 cases. Four of the 5 changes resulted in an increased coincidence with the gold standard diagnosis. One case was a tetanus diagnosed as pneumonia in the MIAb (the patient indeed had a pneumonia, which was considered as a concomitant cause of death), and 2 cases were rabies infections missed in the MIAb. In both cases, the virus was successfully identified in the MIA by polymerase chain reaction (PCR) techniques when the clinical information became available and the Central Nervous System (CNS) samples were secondarily tested. The fourth case was a hereditary factor VIII deficiency clinically diagnosed and missed in the MIAb. Interestingly, in all 4 cases, the addition of clinical data to CDAb resulted in an increased coincidence with the gold standard diagnosis.

In maternal deaths, the addition of clinical information resulted in a change in diagnosis from MIAb to MIAc in 12 (21%) of 57 cases. All changes resulted in an increased coincidence with the gold standard diagnosis. Eleven of the 12 changes in diagnosis were obstetric hemorrhages missed in the MIAb. In 7 of these 12 cases, there was also an increase in accuracy in the CDAb diagnosis.

Finally, in adult deaths, a change in diagnosis from MIAb to MIAc was observed in 13 (12%) of 112 cases. Nine of the 13 changes resulted in an increased coincidence with the gold standard diagnosis. In this group, changes occurred mostly in metabolic conditions (diabetes mellitus) and gastrointestinal disorders. There was another case of rabies, identified also by PCR techniques in the MIA Central Nervous System (CNS) samples once the clinical information triggered the testing. The addition of clinical information resulted in diagnostic accuracy increase of the CDA in 4 of these 13 cases.

### 3.4 Relevant clinical information

Table 2 shows the clinical data that were more informative, resulting in significant changes of the MIAb diagnoses. Evidence of bleeding, underlying chronic conditions, symptoms of gastrointestinal disease, and previous history of accidents or animal bites were particularly instructive.

**Table 2:** The most informative clinical data, resulting in significant changes of the MIAb to clinical data diagnoses

Clinical symptom	No. of cases
Evidence of bleeding	12
Evidence of an underlying chronic condition (eg, hypertension and diabetes)	7
Evidence of gastrointestinal disease (eg, vomiting, diarrhea, and abdominal pain)	5
Evidence of a premortem injury (eg, animal bites)	4
Evidence of a congenital disease (eg, hemoglobinopathies)	1
Recent treatments (eg, antimalarials)	1

## 4 Discussion

This is, to our knowledge, the first study analyzing the contribution of the clinical information to the diagnostic performance of pathologic autopsy methods, namely, the MIA and the CDA, in cause of death determination. Our results show that, although the MIA and the CDA performed and analyzed blindly to any clinical data have a good correlation with the gold standard, the addition of clinical information to the pathological and microbiological findings results in a minor but significant increase in the concordance in the diagnostic group and in higher coincidence (identical *ICD-10* coding in terms of block) between the MIA and the CDA with the gold standard diagnosis (CDA with clinical data). Overall, the addition of clinical data modified the MIAb diagnosis in 35 cases, and in 30 of them (12%), the change resulted in an increased coincidence with the gold standard diagnosis. As a result, the accuracy of the MIA increased from 63% in the MIA blind to clinical information (MIAb) to 76% in the MIA enhanced with clinical information (MIAc).

We have previously reported the validation of the MIA method when compared with the CDA, the gold standard for cause of death attribution [6], [7], [8], [9]. In these studies, we have used a purist approach to specifically determine the validity of the MIA procedure on its own, avoiding the use of clinical information to reach the putative MIA cause of death. The rationale for this was to understand the potential usefulness of the methodology by itself in case it would be applied in sites with minimal or no clinical information available, or at the community level for deaths having occurred in the absence of any contact with the health system. However, the diagnosis of clinical autopsies, since first introduced by the Italian anatomist Giovanni Battista Morgagni more than 250 years ago [18], has traditionally relied on complementing the pathologic observations, initially macroscopic, and after Rudolf Virchow's contributions, also microscopic [19], with any available clinical data. The final diagnosis of a CDA in all pathology departments is based in the precise correlation between the anatomical and pathological findings of the postmortem examination with the premortem clinical information from the patient [18], [20], [21]. Our study shows that the addition of clinical data allowed for obtaining a diagnosis in 6 cases diagnosed as nonconclusive in the CDAb and that this clinicopathological correlation was needed to reach an adequate diagnosis in the CDA in 20 additional cases (8%). Although it may be obvious to accept that the clinical data improve any autopsy results, no studies have evaluated to what extent the contribution of the clinical data is key in the cause of death assignment process.

The present study included different age groups and a wide variety of underlying diseases and syndromes, representative of the wide spectrum of patients attended at a quaternary health care center in a country like Mozambique, and thus, it allows evaluating separately the contribution of this information in all these groups. The improved diagnostic concordance from MIAb to MIAc was particularly high in maternal deaths (*κ* value increasing from moderate to almost perfect agreement, 0.;*P* < .001). Obstetric hemorrhage as a main cause of death was missed in 11 cases in the MIAb (all but 1 case), but was easily identified in the clinical records and, consequently, was correctly diagnosed in the MIA enhanced with clinical data. Interestingly, obstetric hemorrhage was also missed in 7 cases in the CDAb, as no signs of hemorrhage were detected in the autopsy. Because obstetric hemorrhage is a condition easily captured not only in the clinical records but even through a simple interview such as the one conducted in the verbal autopsy [22], [23], [24], our findings indicate that some degree of obstetric information from the clinical records or the verbal autopsy could significantly improve the diagnosis established by the MIA in maternal deaths. The autopsy in women with obstetric hemorrhage has a very limited contribution. However, it may help to exclude other causes of death or other conditions that could have contributed to death, as shown in a previous study conducted by our group in which eclampsia was clinically missed in a patient [25].

In adult deaths, the diagnostic concordance with the gold standard was significantly better with MIAc than with MIAb (*κ* value increasing from substantial agreement in the MIAb to almost perfect agreement in the MIAc, *P* = .021). With the exception of a case of rabies, most of the diseases not diagnosed in the blind MIA and captured in the clinically enhanced MIA were noninfectious in nature, including cardiovascular, metabolic, and gastrointestinal diseases. Diagnosis of such entities, however, remains challenging even with the classical CDA because of the variety of lesions and organs involved. These cases often require a combination of all the available macroscopic data and frequently of the clinical information [26], [27], [28].

The increase in diagnostic concordance from MIAb to MIAc compared with the gold standard was also evident in children, although it did not reach statistical significance. However, the analysis of the cases not diagnosed by the MIAb provided relevant information. Two deaths were secondary to rabies, which was successfully identified by PCR techniques when the clinical data became available and the central nervous system samples were retrospectively tested and consequently correctly diagnosed in the clinically enhanced MIA. Another case was a tetanus misdiagnosed in the blind MIA and correctly diagnosed when the clinical information was available. Again, both rabies and tetanus are conditions that may easily be captured by the clinical history or, in its absence, by a minimum narrative of the signs, symptoms, or events preceding death [29], [30], and consequently, the information obtained through these sources could significantly improve the results of the MIA. Conditions arising from the bites or encounters with insects or animals, or related to intoxications, poisoning, or exposures to other toxins or drugs (including traditional medicines) may present with unspecific systemic changes and thus be difficult to diagnose through the blind observation of tissue samples obtained through the MIA. However, these conditions can easily be suspected or confirmed if the right clues are provided by the clinical or the Verbal Autopsy (VA) interview. Interestingly, in all pediatric cases in which the clinical information contributed to improve the MIA diagnosis, this information was also relevant to amend the CDAb diagnosis.

Finally, in neonates, the improved diagnostic concordance from the MIAb to the MIAc was also significant (*κ* value increasing from moderate to substantial agreement, *P* = .021), although the range of diseases not diagnosed in the MIAb and captured in the clinically enhanced MIA was quite broad and hinders reaching reliable conclusions.

In conclusion, our study indicates that, although the MIA and the CDA blind to any clinical data have a good correlation with the gold standard, the addition of clinical data has a minor but significant impact on this correlation, increasing the diagnostic accuracy of the MIAb and CDAb in 12% and 8% of the cases, respectively. Consequently, the use of clinical data increases the diagnostic precision of the MIA, helping to provide more robust data for cause of death surveillance in resource-limited settings and its collection should therefore be highly encouraged.
